# A cross-sectional study to assess knowledge of women about cervical cancer: an urban and rural comparison

**DOI:** 10.1186/s12199-021-00986-6

**Published:** 2021-06-07

**Authors:** Małgorzata Lesińska-Sawicka

**Affiliations:** Stanislaw Staszic State University of Applied Sciences in Piła, ul. Podchorążych 10, 64-920 Piła, Poland

**Keywords:** HPV, cervical cancer, ubran, rural, knowledge level

## Abstract

**Introduction:**

Cervical cancer and its etiopathogenesis, the age of women in whom it is diagnosed, average life expectancy, and prognosis are information widely covered in scientific reports. However, there is no coherent information regarding which regions—urban or rural—it may occur more often. This is important because the literature on the subject reports that people living in rural areas have a worse prognosis when it comes to detection, treatment, and life expectancy than city dwellers.

**Material and methods:**

The subjects of the study were women and their knowledge about cervical cancer. The research was carried out using a survey directly distributed among respondents and via the Internet, portals, and discussion groups for women from Poland.

Three hundred twenty-nine women took part in the study, including 164 from rural and 165 from urban areas. The collected data enabled the following: (1) an analysis of the studied groups, (2) assessment of the respondents’ knowledge about cervical cancer, and (3) comparison of women’s knowledge depending on where they live.

**Results:**

The average assessment of all respondents’ knowledge was 3.59, with women living in rural areas scoring 3.18 and respondents from the city—4.01. Statistical significance (*p* < 0.001) between the level of knowledge and place of residence was determined. The results indicate that an increase in the level of education in the subjects significantly increases the chance of getting the correct answer. In the case of age analysis, the coefficients indicate a decrease in the chance of obtaining the correct answer in older subjects despite the fact that a statistically significant level was reached in individual questions.

**Conclusions:**

Women living in rural areas have less knowledge of cervical cancer than female respondents from the city. There is a need for more awareness campaigns to provide comprehensive information about cervical cancer to women in rural areas. A holistic approach to the presented issue can solve existing difficulties and barriers to maintaining health regardless of the place of life and residence.

**Implication for cancer survivors:**

They need intensive care for women’s groups most burdened with risk factors.

## Introduction

Cervical cancer and its etiopathogenesis, the age of women in whom it is diagnosed, average life expectancy, and prognosis are information widely covered in scientific reports [1–7]. However, there is no coherent information regarding which regions—urban or rural—it may occur more often. This is important because the literature on the subject reports that people living in rural areas have a worse prognosis when it comes to detection, treatment, and life expectancy than city dwellers. The literature on the subject shows a clear disproportion in the survival rate of rural residents who have been diagnosed with cancer in comparison to people living in cities [8–15].

Apart from access to health care, environmental factors, and genetic conditions, the state of health is significantly influenced by lifestyle which, among other factors, is associated with knowledge about health and healthcare. A population’s medical knowledge is one of the determinants of attitudes and behaviour related to maintaining health and preventing disease. The level of medical knowledge depends not only to a large extent on proper self-care in health and illness, but also on the awareness of the need to observe one’s own body and limit risky or anti-health activities that may have a detrimental effect.

Cervical cancer is a malignant tumour that develops from the epithelium covering the cervix or cervical canal. Worldwide, 500,000 cases of cancer are diagnosed each year, and more than half of those affected die. It is estimated that by 2050, the number of cases could reach over 1 million per year [16]. Globally, 80% of new cervical cancer cases occur in developing countries, where it ranks first as a cause of death among women compared to other genital cancers. Declining trends in incidence and low mortality in developed countries are associated with the implementation of universal and continuous prevention and with improved access to the health care system [17].

Approximately 570,000 cases of cervical cancer and 311,000 deaths from the disease occurred in 2018. Cervical cancer was the fourth most common cancer in women, ranking after breast cancer (2.1 million cases), colorectal cancer (0.8 million), and lung cancer (0.7 million). The estimated age-standardised incidence of cervical cancer was 13.1 per 100,000 women globally and varied widely among countries, with rates ranging from less than 2 to 75 per 100,000 women [17, 18].

According to Arbyn et al. globally in 2018, the average age at diagnosis of cervical cancer was 53 years, ranging from 44 years (Vanuatu) to 68 years (Singapore). The global average age at death from cervical cancer was 59 years, ranging from 45 years (Vanuatu) to 76 years (Martinique). Cervical cancer ranked in the top three cancers affecting women younger than 45 years in 146 (79%) of 185 countries assessed [18].

Cervical cancer is a special problem, both medically and socially. Medically, because despite the changes in treatment methods and the increasing availability of preventive examinations, a large number of women are still diagnosed with advanced stages of cancer, socially because most often this disease affects women between 35 and 55 years of life, i.e. young and middle-aged women who are professionally active and perform a number of social roles. Knowledge of risk factors, risk behaviour, unspecific symptoms of cervical cancer, and ways of preventing the onset of the disease are the basic elements of health knowledge, which are intended to protect against its occurrence and/or to diagnose it quickly and enable early treatment. This will help to early detection, improve prognosis, and reduce death rates.

## Objective

The starting point for the study were scientific reports on inequalities in access to health care for rural residents.

The aim of the study is to assess the relationship between the level of knowledge of women about cervical cancer and their place of residence.

This is important because literature on the subject reports that people living in rural areas have a worse prognosis when it comes to detection, treatment, and life expectancy than city dwellers. This may be related to the level of knowledge. An assessment of women’s knowledge of cervical cancer, depending on where they live, may allow appropriate educational programmes to be adapted, raising the level of knowledge and thus reducing the risk of the disease.

## Material and methods

### Study design

The presented research is a cross-sectional and comparative study. The study was carried out using the diagnostic survey method, using the author’s own anonymous questionnaire.

The survey proper was preceded by a pilot survey outside the study area. The survey questionnaires were distributed to 20 women who came for preventive examinations at the gynaecological surgery in the month of November 2018. After the respondents answered the questions, the survey questionnaires were transferred to a specially prepared box located at the surgery registration desk. Based on the feedback received from the pilot study, the construction of the survey questionnaire was completed. Several questions were modified in terms of clarity and precision of wording.

The proper questionnaire contained 14 questions. Of the 14 questions, 11 concerned issues of reproductive organ physiology, cervical cancer risk factors, causes, symptoms, and preventive measures and 3 were metric questions.

### Participants

The study involved 329 women living in rural and urban areas and their knowledge about cervical cancer. When selecting respondents, their place of residence was taken into account.

### Data collection

The research was conducted from December 2018 to June 2019. Before completing the questionnaire, respondents were obliged to declare their consent to participate in the study. The research was carried out using a survey via the Internet, portals, and discussion groups for women from Poland. Three hundred and fifty questionnaire forms were distributed. The analysis was based on 329 correctly completed surveys. The collected data enabled the following: (1) analysis of the studied groups, (2) assessment of the respondents’ knowledge about cervical cancer, and (3) comparison of women’s knowledge depending on where they live.

### Data analysis

A chi-square test was used. Acceptable probability p (level of significance of the test) was assumed to be *p* ⩽ 0.05. Logistic regression models were used to estimate odds ratios (ORs) between four levels of knowledge and place for living with a 95% confidence interval. ORs were estimated with adjustment for age and education level which were selected on the basis of their bivariate association with answers on questions. The distributions of the variables were characterised by the following: group size (N), arithmetic mean (X̅), and significance level (p). Statistical analysis was performed using the STATISTICA PL version 10.

The respondents’ answers were verified and assessed on a scale:

**Table:** 

Percentage of correct answers	Qualitative evaluation of knowledge	Quantitative evaluation of knowledge
90–100%	Very good	5
70–89%	Good	4
50–69%	Sufficient	3
49% and less	Insufficient	2

The study was approved by the Bioethics Committee.

## Results

Three hundred twenty-nine women took part in the study, including 164 from rural and 165 from urban areas. The age of the respondents mostly ranged between 21 and 30 years (urban—56.4%, rural—48.8%). The study participants generally had secondary education (city—53.7%, village—51.5%) (Table 1).

**Table 1:** The characteristics of the examined population

Indicator	Women from rural areas ***N*** = 164 (%)	Women from urban areas ***N*** = 165 (%)	Statistical significance
**Age (in years)**			
≤ 20	13.4	13.4	***p*** **= 0.00077**
21–30	48.8	56.4	
31–40	17.1	14.8	
41–50	17.1	12.4	
≥ 51	3.6	3.0	
**Education level**			
Elementary	3.5	1.2	***p*** **= 0.00000**
Vocational	9.5	2.4	
Secondary	51.5	53.7	
Higher	35.5	42.7	

The respondents participating in the study were evaluated in terms of their basic knowledge about cervical cancer (Table 2). Women living in the city answered the questions correctly more often than women from rural areas. A statistical significance was found between the place of residence and the respondents’ knowledge mainly in terms of: knowledge of precancerous conditions (question 2; *p* = 0.019), causes of cervical cancer (question 4; *p* < 0.001), symptoms of cervical cancer in the initial stage (questions 6–7; *p* = 0.001, *p* < 0.001), cytological examination (questions 8–10; *p* < 0.001), and initial treatments (*p* = 0.010).

**Table 2:** Summary of questions and percentage of correct answers from the questionnaire with the number of respondents and the breakdown by village and city

Answer/correct answer	Number of correct answers from women from rural areas ***N*** = 164 (%)	Number of correct answers from women from urban areas ***N*** = 165 (%)	Statistical significance
**Question 1.** The cervix is (choose the correct statement): **Answer:** Exposed to the occurrence and development of infection	63.4	93.9	*p* = 0.392
**Question 2**. Pre-cancerous conditions of cervical cancer include (choose the correct statement): **Answer:** Visible changes of epithelial cells under microscopic examination crossing the border of the basal membrane	56.1	57.3	***p*** **= 0.019**
**Question 3.** The risk factors for cervical cancer include (choose the correct statement): **Answer:** Smoking cigarettes	24.3	6.1	*p* = 0.683
**Question 4.** The most common causes of cervical cancer include (choose the correct statement): **Answer:** HPV infection	53.7	80.5	***p*** **< 0.001**
**Question 5.** The main route of HPV infection is: **Answer:** Vaginal sexual intercourse	67.1	87.8	*p* = 0.508
**Question 6.** What is the common initial symptom of cervical cancer (choose the correct statement): **Answer:** Irregular intermenstrual bleeding	51.2	58.5	***p*** **= 0.001**
**Question 7.** Cervical cancer (choose the correct statement): **Answer:** At the initial stage it is asymptomatic	62.2	85.4	***p*** **< 0.001**
**Question 8.** To reduce the risk of full-blown cervical cancer you should (choose the correct statement): **Answer**: Perform regular cytological tests, at least once every 3 years	67.1	97.6	***p*** **< 0.001**
**Question 9.** The basic prophylactic examination of cervical cancer is (choose the correct statement): **Answer:** A cytological examination	65.9	90.2	***p*** **< 0.001**
**Question 10**. Cytological examination is (choose the correct statement): **Answer:** An assessment of cell smear taken from the canal and cervical disc	68.3	97.6	***p*** **< 0.001**
**Question 11.** Initial treatment for cervical cancer is (choose the correct statement): A**nswer:** Local removal of cancerous lesions	62.2	64.1	***p*** **= 0.010**

Based on the answers received, the level of knowledge of the respondents was assessed (Table 3). The average assessment of all respondents’ knowledge was 3.59. Women living in rural areas scored 3.18, while urban respondents achieved a higher average of 4.01. Statistical significance (*p* < 0.001) between the level of knowledge and place of residence was found.

**Table 3:** Assessment of the level of knowledge of the respondents according to their place of residence

Assessment of the level of knowledge	Women from rural areas ***N*** = 164	Women from urban areas ***N*** = 165	Statistical significance
Very good	11.0%	29.3%	***p*** **< 0.001**
Good	34.1%	47.6%	
Sufficient	17.1%	18.3%	
Insufficient	37.8%	4.8%	
X̅	3.18	4.01	

An analysis was performed using logistic regression for the dependent variable—cervical cancer knowledge and the independent variables—age and education (Table 4).

**Table 4:** Summary of logistic regression models for answers from the questionnaire (0—false, 1—true) as a dependent variable and education level and age as independent variables

Answer/correct answer	Education level		Age	
	OR	Statistical significance	OR	Statistical significance
**Question 1.** The cervix is (choose the correct statement): **Answer:** Exposed to the occurrence and development of infection	3.45	***p*** **< 0.001**	0.44	***p*** **< 0.001**
**Question 2**. Pre-cancerous conditions of cervical cancer include (choose the correct statement): **Answer:** Visible changes of epithelial cells under microscopic examination crossing the border of the basal membrane	1.25	*p* = 0.25	1.21	*p* = 0.29
**Question 3.** The risk factors for cervical cancer include (choose the correct statement): **Answer:** Smoking cigarettes	0.7	*p* = 0.17	1.5	***p*** **= 0.07**
**Question 4.** The most common causes of cervical cancer include (choose the correct statement): **Answer:** HPV infection	1.9	***p*** **= 0.002**	0.93	*p* = 0.69
**Question 5.** The main route of HPV infection is: **Answer:** Vaginal sexual intercourse	2.57	***p*** **< 0.001**	0.63	***p*** **= 0.025**
**Question 6.** What is the common initial symptom of cervical cancer (choose the correct statement): **Answer:** Irregular intermenstrual bleeding	1.6	***p*** **= 0.02**	0.84	***p*** **= 0.34**
**Question 7.** Cervical cancer (choose the correct statement): **Answer:** At the initial stage it is asymptomatic	2.67	***p*** **< 0.001**	0.54	***p*** **= 0.003**
**Question 8.** To reduce the risk of full-blown cervical cancer you should (choose the correct statement): **Answer**: Perform regular cytological tests, at least once every 3 years	3.81	***p*** **< 0.001**	0.59	***p*** **= 0.02**
**Question 9.** The basic prophylactic examination of cervical cancer is (choose the correct statement): **Answer:** A cytological examination	3.03	***p*** **< 0.001**	0.83	***p*** **= 0.37**
**Question 10**. Cytological examination is (choose the correct statement): **Answer:** An assessment of cell smear taken from the canal and cervical disc	5.1	***p*** **< 0.001**	0.45	***p*** **< 0.001**
**Question 11.** Initial treatment for cervical cancer is (choose the correct statement): A**nswer:** Local removal of cancerous lesions	3.27	***p*** **< 0.001**	0.66	***p*** **= 0,04**

The results indicate that an increase in the level of education in the subjects significantly increases the chance of getting the correct answer. In the case of age analysis, the coefficients indicate a decrease in the chance of obtaining the correct answer in older subjects despite the fact that a statistically significant level was reached in individual questions. This may indicate a complex dependence of age on the outcome of obtaining the correct answer.

## Discussion

Scientific reports indicate that women have insufficient knowledge about cervical cancer. The scale of the phenomenon shows that this is a problem that transcends national and continental borders [19–23].

Having basic medical knowledge is of great importance in the prevention of cervical cancer. It facilitates early detection of the disease, obtaining information on the state of health and taking early therapeutic measures [24].

The respondents of the presented study most often knew the correct answers to questions about the initial methods of treating cervical cancer (82.9% correct answers), the importance of cytological tests in the prevention of cervical cancer (82.3%), the characteristics of cytological tests (78.0%), and methods of HPV infection (77.4%). The results obtained coincide with reports by other authors who drew attention to the fact that women know the importance of cytological tests in prophylaxis and the role of HPV in the aetiology of cervical cancer [25].

However, it is worrying that women know little about risk factors, precancerous conditions, or the initial symptoms of cervical cancer. Having knowledge of the risk factors or early symptoms of cervical cancer means that women may be able to recognise abnormalities and seek medical help. Also, when women are aware of the causes and risk factors of cervical cancer and perceive themselves to be at risk, they are more likely to take up measures to prevent the acquisition of the human papillomavirus hence avoid developing the disease [26]. Other studies have shown that awareness of cervical cancer symptoms and prevention measures and perception of being at risk of the disease were associated with intention to go for screening and thus its early detection [27, 28].

Due to the fact that the general level of knowledge about cervical cancer was found to be unsatisfactory among the respondents, despite individual correct answers, the author of this study decided to additionally check whether the place of residence potentially exacerbates the already insufficient level of medical knowledge.

In a few reports on the impact of women’s residence on their level of knowledge about cervical cancer, there is no agreement on the issue. In studies conducted by Saweryn and Wróbel among high school students, no difference was found between the level of girls’ knowledge regarding risk factors for cervical cancer and their place of residence [29]. At the same time, Warzecha et al. examined 2000 women on their knowledge about the physiology of the menstrual cycle, methods of contraception, infertility, and prevention of cervical cancer, and came to different conclusions. They proved that residence in an urban area, inter alia, had a significant impact on the number of correct answers (*p* = 0.002). They also came to an important conclusion about improving the level of women’s knowledge about reproduction, especially among women with a lower level of education and living in small communities [30]. Similarly, Sulima et al. found a statistically significant correlation between the knowledge of the studied women about early sexual intercourse as a factor risk of cervical cancer and place of residence (*p* = 0.01) [31]. This is also confirmed by Reks’ research, which indicates more knowledge about cervical cancer among women living in cities [32].

Based on the results obtained and the cited reports, it can be presumed that factors affecting the difference in knowledge level appear at a later stage in women’s lives (the level of knowledge of high school students in studies by Saweryn and Wróbel did not depend on the place of residence). In Polish schools, in selected classes, issues related to hygiene, preventive examinations, and risk factors of the most common diseases are raised. Young women leaving high school are equipped with basic medical knowledge: their fate, paths, and life choices to either deepen this knowledge, supplement it, or completely forget it.

The lack of a sufficiently high level of medical knowledge is a factor perceived by various authors as a barrier to adequate self-observation, self-care, and undertaking of activities related to the prevention of disease occurrence, the early recognition of risks, and the implementation of therapeutic measures [33, 34].

## Limitations

The study, although providing important knowledge, is subject to several methodological limitations common to small observational studies.

Conducting the research via the Internet resulted in limited access to respondents. Only people who participated in discussion groups on social networking sites and were interested in the topic took part. This may give an inaccurate picture of the knowledge of women from urban and rural areas. The results cannot be generalised to all women living in those communities. Moreover, because of the nature of the cross-sectional design of this study, the author is not able to determine the cause-effect association for our findings.

The author of the study is aware that the level of knowledge is influenced by many factors, not always related to the place of residence. Nevertheless, in view of scientific reports of higher morbidity and mortality due to cancer among people from rural areas, attention was paid to the educational aspect, which among other factors is related to inequalities in access to health care and can be solved at the level of school education and continued at a later time.

Furthermore, the declared knowledge of female respondents does not always translate into health-seeking behaviour, which was not taken into account in this study. Despite these limitations, the current study provides precious information regarding Polish women.

## Conclusions

Women living in rural areas have less knowledge of cervical cancer than female respondents from the city. Additionally, knowledge is influenced by education level and age. The better educated a woman is the more likely she was to have more general knowledge. The older a woman is, the lower her level of knowledge.

In summary, it can be said that the older a woman is, from a rural area with a lower level of education, the worse her medical knowledge. Research on women’s medical knowledge should form the basis for health education in society, implemented not only by medical institutions but also by schools at all levels of education. It is important to reduce the differences in access to medical professionals—gynaecologists, midwifes, or nurses—for the issue discussed here and to organize work in such a way that the promotion of health for gynaecological patients is the core of further medical interventions. It is important to reach out to women in different ways and to not stop educating women after they finish their education.

Additional research is required to further understand and assess the effectiveness of different strategies to increase cervical cancer knowledge.

The differences in the level of knowledge among women living in urban and rural areas should be discussed in various sectors of social life. There is also a need for more awareness campaigns to provide comprehensive information about cervical cancer to women in all areas and dispel any negative beliefs and perceptions. A holistic approach to the presented issue can solve existing difficulties and barriers to maintaining health regardless of the place of life and residence.

## Data Availability

All data in the study can be accessed from the corresponding author upon request.

## Abbreviations

HPV: Human papilloma virus
